# Incidence and Factors Associated with Suboptimal Exercise Workload Progression in Cardiac Rehabilitation: A Retrospective Cohort Study

**DOI:** 10.3390/jcm15197713

**Published:** 2026-10-06

**Authors:** Guy Halevi, Oren Caspi, Noa Haimovich, Omer Golan, Noa Rentsler, Barak Zafrir

**Affiliations:** 1 Department of Cardiology, Rambam Health Care Campus, Haifa 3109601, Israel; 2 The Ruth and Bruce Rappaport Faculty of Medicine, Technion Israel Institute of Technology, Haifa 3525433, Israel

**Keywords:** cardiac rehabilitation, metabolic equivalents, exercise workload, cardiorespiratory fitness

## Abstract

**Background:** Improving cardiorespiratory fitness is a central goal of cardiac rehabilitation (CR), yet exercise workload progression varies substantially. We aimed to characterize changes in treadmill workload during CR and determine the frequency of, and the factors associated with, suboptimal progression. **Methods:** This retrospective cohort study included 180 patients enrolled in an outpatient CR program. Treadmill exercise workload was quantified in metabolic equivalents (METs) from recorded speed and grade using estimated oxygen-consumption equations. Using prespecified exploratory thresholds, suboptimal progression was defined as an absolute increase of <1 MET or a relative increase of <30%, determined by comparing mean workloads achieved during the initial and final four sessions. Factors associated with suboptimal progression were assessed using multivariable logistic regression. **Results:** Median workload increased from 3.07 METs (IQR 2.40–4.01) during the initial four sessions to 3.90 METs (IQR 2.93–5.56) during the final four sessions (*p* < 0.001), with improvements observed across baseline exercise capacity, age, and sex strata. Nevertheless, 104 patients (57.8%) achieved <1 MET improvement and 97 (53.9%) achieved <30% relative improvement. In the primary multivariable analyses, female sex and higher body mass index were associated with suboptimal progression under both definitions; a history of coronary artery disease was additionally associated with suboptimal absolute progression, and recent smoking with suboptimal relative progression. Among concurrent training-related factors, lower session attendance was associated with both definitions, whereas higher mean perceived exertion was associated with suboptimal relative progression. **Conclusions:** More than half of participants demonstrated suboptimal treadmill workload progression despite significant overall improvement during CR. Routine monitoring of session-level workload may facilitate identification of patients with limited progression and support individualized adjustment of exercise prescriptions.

## 1. Introduction

Cardiac rehabilitation (CR) is a cornerstone of secondary prevention and is associated with improved exercise capacity, quality of life, and long-term clinical outcomes [1]. Cardiorespiratory fitness is an important therapeutic target, as each 1-metabolic equivalent (MET) increase in fitness has been associated with a 13–15% reduction in all-cause mortality and cardiovascular events [2,3]. Accordingly, meaningful gains in exercise capacity are central to optimizing CR outcomes.

Progressive increases in exercise workload provide the training stimulus required to improve cardiorespiratory fitness [4]. In supervised outpatient CR, treadmill workload can be quantified during exercise sessions in METs from speed and grade using established American College of Sports Medicine (ACSM) equations [5]. Although CR generally improves exercise capacity, substantial interindividual variability exists in workload progression and training response [6,7]. Most studies assess improvement by comparing peak oxygen consumption or METs before and after CR [8,9,10,11]. However, early post-event or post-surgical testing may be limited by symptoms, pain, kinesiophobia, or conservative protocols and may not fully reflect training adaptation [12].

Session-level treadmill workload reflects achieved training intensity and is distinct from cardiorespiratory fitness or peak exercise capacity measured during formal exercise testing. Despite its clinical importance, session-by-session workload progression in routine CR remains poorly characterized. Exercise prescription commonly relies on baseline testing and patient-reported rating of perceived exertion (RPE) [13], while characteristics associated with inadequate workload progression are not well established [14,15,16]. These gaps may contribute to variability in CR effectiveness.

We therefore aimed to characterize treadmill workload progression during supervised outpatient routine CR sessions, determine the incidence of suboptimal progression using prespecified absolute (<1 MET) and relative (<30%) thresholds, and evaluate baseline demographic and clinical characteristics and concurrent training-related factors associated with suboptimal progression.

## 2. Materials and Methods

### 2.1. Study Population

This retrospective cohort included consecutive patients participating in outpatient CR at Rambam Health Care Campus (Haifa, Israel) between 1 March and 31 December 2025. The multidisciplinary program comprised twice-weekly 1.5 h supervised sessions including aerobic and resistance exercise, nutritional counseling, risk-factor management, and psychosocial support. Patients could complete up to 24 sessions over 3 months; those attending <16 sessions or unable to exercise on a motorized treadmill were excluded (Figure 1). The study was approved by the Rambam Health Care Campus Institutional Ethics Committee (0398-25-RMB-D) and conducted in accordance with the Declaration of Helsinki; informed consent was waived because of the retrospective design.

### 2.2. Study Variables and Definitions

Demographic and clinical data were retrieved from electronic medical records. Patient-reported and performance measures were routinely assessed at CR admission. Self-perceived health was measured by the EQ Visual Analogue Scale (EQ-VAS; poor health <50), anxiety and depression by the GAD-7 and PHQ-9 (clinically significant symptoms ≥10), and handgrip strength by dynamometry (muscle weakness < 27 kg in men and <16 kg in women).

### 2.3. Exercise Prescription and Workload

Exercise capacity at CR admission was assessed by symptom-limited treadmill testing to determine peak METs. Exercise prescriptions followed FITT-VP principles [13]. Intensity was based on heart-rate reserve (40–70%), RPE, and baseline functional capacity, with a target Borg RPE of 12–14 [17]. Sessions included continuous treadmill walking or running for up to 20 min, which was obligatory in all attended sessions and constituted the primary aerobic modality. Bicycle, elliptical, or rowing exercise was used less commonly as additional aerobic training according to patient preference and physical limitations and was not included in the workload analysis. Training heart-rate ranges were updated every 4–6 sessions, with progression guided by symptoms and target intensity.

Training exercise workload was quantified from the treadmill speed and grade recorded during each rehabilitation session, calculated according to ACSM equations for estimated oxygen consumption (VO_2_) [5]. For walking, VO_2_ = 3.5 + horizontal component speed × 0.1 + vertical component speed × grade × 1.8; for running, VO_2_ = 3.5 + horizontal component speed × 0.2 + vertical component speed × grade × 0.9, where speed is expressed in m/min and grade is expressed as a decimal fraction. METs were calculated as VO_2_ divided by 3.5.

Each participant received an individualized exercise-training card. At CR initiation, an exercise physiologist instructed patients how to record treadmill speed, grade, exercise duration, and perceived exertion using the Borg RPE scale. During each supervised session, patients recorded the treadmill settings actually performed, exercise duration, and RPE. Exercise physiologists and CR trainers were present on the training floor throughout the sessions, assisted with card completion when needed, and routinely checked the recorded values for completeness and consistency with the supervised exercise session. The recorded training data and exercise prescription were formally reviewed by an exercise physiologist every 4–6 sessions and modified as clinically appropriate. Patient self-recording was incorporated as part of the program’s self-management and self-efficacy approach. Following a brief warm-up, treadmill exercise was continuous, and speed and grade were maintained at the prescribed training level for most of the exercise bout; these steady training values were used to estimate session workload. The walking or running ACSM equation was selected by the supervising CR physiologist according to the exercise mode performed. No session-level values required for the workload calculation were missing in the analyzed sessions, and no imputation was performed.

The primary measure was change in treadmill workload between mean METs during the initial and final four CR sessions. Averaging four sessions at each time point was intended to reduce session-to-session variability and reflect sustained workload rather than an isolated measurement. Prespecified exploratory definitions of suboptimal progression were an absolute increase <1 MET or a relative increase <30%, calculated as [(final mean METs—initial mean METs)/initial mean METs] × 100. The 30% threshold was selected a priori as an exploratory proportional benchmark to complement the absolute 1-MET criterion and account for differences in baseline workload; relative improvements of approximately 30–45% have been reported in previous CR cohorts and subgroups [8]. It was not intended as a validated clinical cut-off.

### 2.4. Data Analysis

Continuous variables are presented as mean ± SD or median (IQR), and categorical variables as frequencies and percentages. Groups were compared using Student’s *t*-test or Mann–Whitney U test for continuous variables and chi-square or Fisher exact test for categorical variables, as appropriate. Initial and final mean METs were compared using the Wilcoxon signed-rank test.

Associations of baseline demographic and clinical characteristics and concurrent training-related factors with suboptimal progression were examined using univariable and multivariable logistic regression. Candidate variables were considered based on clinical relevance and availability in routine CR practice, with univariable *p* < 0.15 used as a screening criterion for multivariable consideration. Separate models were constructed for each progression definition. Hierarchical models evaluated the additional contribution of admission peak METs and concurrent training-related factors, including mean RPE and session attendance. Multicollinearity was assessed by correlation matrix; model fit and explanatory power by omnibus tests and Nagelkerke R-squared; calibration by the Hosmer–Lemeshow test; and apparent discrimination within the study sample by the area under the receiver operating characteristic curve (AUC). The models were intended to examine adjusted associations rather than to develop a clinical prediction tool; therefore, AUC values were considered descriptive model characteristics and were not internally validated.

As a sensitivity analysis, the final multivariable models were additionally adjusted for mean treadmill workload during the initial four sessions. To address the potential influence of varying numbers of attended sessions on workload progression, a second sensitivity analysis used a standardized session interval common to all participants, comparing mean treadmill workload during sessions 1–4 with mean workload during sessions 13–16. The same absolute and relative progression thresholds were applied, and the multivariable models were repeated using the covariates included in the primary models, except total session attendance, which was omitted because all participants had completed 16 sessions at the standardized endpoint; for this analysis, mean RPE was calculated over sessions 1–16.

Statistical significance was set at *p* < 0.05. Statistical analyses were performed using SPSS software (Version 25; IBM Corp., Armonk, NY, USA).

## 3. Results

### 3.1. Baseline Characteristics

The study cohort included 180 patients with a median age of 67 years (IQR, 57–73), of whom 34 were women (18.9%). Common comorbidities included hypertension in 113 patients (62.8%), diabetes mellitus in 66 patients (36.7%), and obesity in 47 patients (26.1%). The most frequent indications for CR were myocardial infarction (38.3%), recent cardiac surgery (37.2%), and heart failure (17.8%). The median number of attended sessions was 24 (range 16–24), and the mean RPE reported during CR sessions was 12.4 ± 1.3. Patient characteristics stratified according to absolute and relative workload-progression thresholds are presented in Table 1. Patients with suboptimal absolute workload progression were more frequently women, had higher BMI, and had higher rates of chronic kidney disease, prior coronary artery disease, and heart failure. They also reported higher average RPE during CR sessions. Using the relative threshold, patients with suboptimal progression were more frequently women and had higher BMI.

Patient-reported and functional impairments were also common: 25.6% of patients had an EQ-VAS score < 50, 25.6% had GAD-7 ≥ 10, 30% had PHQ-9 ≥ 10, and 32.8% had low handgrip strength. None of these measures differed significantly between patients with and without suboptimal workload progression according to either the absolute or relative definition.

### 3.2. Exercise Workload Progression

Overall, exercise workload increased significantly during CR. Median workload rose from 3.07 METs (IQR, 2.40–4.01) during the initial four sessions to 3.90 METs (IQR, 2.93–5.56) during the final four sessions (*p* < 0.001). Significant improvements were observed across all baseline exercise-capacity strata, including patients with admission peak METs <5, 5–8, and >8 METs (Table 2). Similar improvements were observed when patients were stratified by sex and by median age (<67 vs. ≥67 years) (Figure 2).

### 3.3. Incidence of Suboptimal Workload Progression

Despite the overall improvement in training workload, suboptimal progression was common. Using the absolute threshold, 104 patients (57.8%) did not achieve an improvement of ≥1 MET. Using the relative threshold, 97 patients (53.9%) did not achieve an improvement of ≥30%. Rates of suboptimal progression were lower among patients with higher baseline exercise capacity, particularly among those achieving >8 METs on admission exercise testing (Table 2). Detailed case descriptions presented in the Supplementary Data further illustrate the marked interindividual variability in exercise workload progression during CR. Selected patients with complex clinical profiles showed suboptimal absolute and/or relative workload improvements, whereas others, including patients recovering from uncomplicated cardiac surgery, demonstrated favorable progression trajectories. These examples highlight the potential value of interpreting workload progression in relation to the patient’s baseline clinical profile and initial exercise capacity.

### 3.4. Factors Associated with Suboptimal Workload Progression

In univariable logistic regression analyses, female sex, higher BMI, chronic kidney disease, history of coronary artery disease, heart failure, and higher average RPE were associated with greater odds of suboptimal absolute progression (Table 1 and Table 3). For the relative threshold, female sex and higher BMI were significantly associated with suboptimal progression. Recent smoking, obesity, cardiac surgery, session attendance, and average RPE met the prespecified criterion for multivariable consideration.

In multivariable logistic regression, baseline characteristics associated with suboptimal relative progression (<30%) after adjustment were female sex, higher BMI, and recent smoking; concurrent training-related factors associated with this outcome were higher mean RPE and lower session attendance (Table 4). Female sex was associated with 2.64-fold higher odds of suboptimal relative progression, while each 1 kg/m^2^ increase in BMI was associated with an 11% increase in odds. Each additional attended CR session was associated with lower odds of suboptimal relative progression. For the absolute threshold (<1 MET improvement), baseline characteristics associated after adjustment were female sex, higher BMI, and history of coronary artery disease, while lower session attendance was the concurrent training-related factor associated with suboptimal absolute progression. No relevant multicollinearity was observed among admission peak METs, average RPE, and session attendance (r < 0.2). The apparent AUCs within the study sample were 0.737 (95% CI, 0.664–0.810) for the relative-threshold model and 0.754 (95% CI, 0.683–0.825) for the absolute-threshold model. These values are reported descriptively as characteristics of model discrimination and were not internally validated.

Additional adjustment for initial four-session treadmill workload did not materially alter the observed associations (Supplementary Table S1). To address the differing opportunity for workload progression associated with variable total session attendance, a sensitivity analysis standardized the assessment interval by comparing mean treadmill workload during sessions 1–4 with that during sessions 13–16 in all participants. In the relative-progression model, higher BMI (adjusted OR 1.09, 95% CI 1.01–1.17; *p* = 0.025) and higher mean RPE (adjusted OR 1.36, 95% CI 1.05–1.76; *p* = 0.020) remained significantly associated with suboptimal progression, whereas female sex and recent smoking were not statistically significant. In the absolute-progression model, history of coronary artery disease remained significantly associated with suboptimal progression (adjusted OR 3.90, 95% CI 1.51–10.05; *p* = 0.005). The point estimates for female sex remained above unity in both models, while the association with BMI in the absolute model was similar in magnitude to the primary analysis but did not reach statistical significance (Supplementary Table S2).

## 4. Discussion

In this retrospective cohort study of patients participating in a multidisciplinary outpatient CR program, treadmill-derived exercise workload increased significantly over the course of rehabilitation. This favorable trajectory was observed consistently across baseline functional-capacity strata, age categories, and sex, demonstrating improvement in treadmill training workload under real-world clinical conditions. These findings are also concordant with previously reported patterns of workload progression in CR [18,19,20,21]. Nevertheless, despite this overall improvement, more than half of the cohort did not achieve predefined thresholds for adequate workload progression, defined as either a ≥1-MET absolute increase or a ≥30% relative increase between the initial and final four training sessions. In the primary multivariable analyses, baseline characteristics associated with suboptimal progression included female sex, higher BMI, recent smoking, and history of coronary artery disease, while lower attendance and higher mean RPE were concurrent training-related factors. These characteristics may help identify patients who require closer monitoring and more individualized exercise progression during CR.

Baseline patient-reported and functional measures also demonstrated a substantial burden of impairment in this CR population. Poor self-perceived health (EQ-VAS < 50) and clinically significant anxiety symptoms (GAD-7 ≥ 10) were each present in 25.6% of patients, clinically significant depressive symptoms (PHQ-9 ≥ 10) in 30%, and low handgrip strength in 32.8%. However, none of these measures was significantly associated with suboptimal workload progression according to either the absolute or relative definition in univariable analyses. These findings suggest that, although functional strength and psychosocial health represent important dimensions of the CR population, they did not distinguish patients with limited treadmill workload progression in the present cohort.

The clinical relevance of exercise workload progression during CR is supported by prior studies showing that cardiorespiratory fitness is strongly associated with long-term prognosis, with each 1-MET increase linked to meaningful reductions in mortality and cardiovascular events [3]. Previous research emphasizes the prognostic importance of achieving adequate workload benchmarks. Patients who exit CR exercising below 3.5 METs are at a substantially higher risk for both all-cause mortality and heart failure hospitalization [18,19]. A functional capacity of approximately 4 METs is generally sufficient for most basic home activities of daily living and light household tasks, whereas higher-capacity activities such as stair climbing, carrying groceries, and heavier housework typically require approximately 5–6 METs [22]. Of note, functional capacity of <4 METs was shown to be associated with increased risk for perioperative cardiac events, regardless of whether the limitation was due to coronary artery disease, heart failure, or a non-cardiac condition [23].

While many CR studies evaluate improvement using exercise tests performed before and after the program, our approach focused on training workload achieved during routine CR sessions. Session-level treadmill speed and grade are routinely recorded during CR and can be translated into estimated METs using established ACSM equations [5], providing a practical measure of training workload progression. However, this measure should not be interpreted as a direct assessment of change in peak VO_2_ or cardiorespiratory fitness. Averaging workload during the first and last four sessions reduces the effect of session-to-session variability and may better reflect sustained workload progression than a single measurement. The finding that over half of participants met the exploratory criteria for suboptimal workload progression highlights substantial interindividual variability in workload advancement during routine CR.

The finding that suboptimal workload progression was less frequent among patients with higher baseline exercise capacity (>8 peak METs) contrasts with previous evidence suggesting that individuals with lower baseline fitness generally achieve greater relative gains in fitness after exercise training [24,25]. This may be explained by the greater physiological reserve of higher-fit patients (especially when comparing heart failure and non-heart failure patients), fewer exercise-limiting symptoms, and a greater ability to tolerate higher training intensities. In contrast, patients with lower baseline capacity may require more individualized, tailored progression strategies, dedicated programs according to etiology and baseline METs, closer monitoring, and potentially longer or more gradual CR programs to achieve clinically meaningful gains.

Female sex was associated with suboptimal workload progression under both the absolute and relative definitions after multivariable adjustment. Women had more than threefold higher odds of failing to achieve a 1-MET improvement and more than twofold higher odds of failing to achieve a 30% relative improvement. This finding is consistent with previous studies reporting smaller absolute gains in fitness among women and lower training responses even after adjustment for relevant covariates [8,9]. Similarly, data from the Henry Ford CR experience showed that women achieved lower mean absolute MET improvements after CR compared to men (0.9 METs vs. 1.3 METs) [20]. Several mechanisms may contribute to this association, including lower baseline muscle mass, greater symptom burden, musculoskeletal limitations, sex-related differences in exercise prescription or training intensity, and barriers to optimal participation. These findings suggest that women participating in CR may benefit from closer monitoring of workload progression, more individualized intensity adjustment, and targeted attention to barriers that may limit progression. However, given the relatively small number of women in the present cohort, this association should be interpreted cautiously and requires confirmation in populations with greater female representation.

Higher BMI was associated with both progression definitions after multivariable adjustment; each 1 kg/m^2^ increase was associated with 11% higher odds of suboptimal relative and 8% higher odds of suboptimal absolute progression. This may reflect reduced aerobic efficiency, orthopedic limitations, dyspnea, or greater perceived exertion. Exercise prescriptions for patients with obesity may need to balance tolerability with adequate progression, incorporating alternative aerobic modalities, resistance training, and weight-management support [26].

Recent smoking and history of coronary artery disease were associated with suboptimal relative and absolute progression, respectively, after multivariable adjustment, and may reflect different dimensions of patient vulnerability. Recent smoking may indicate poorer baseline conditioning, respiratory limitations, ongoing vascular risk, or lower engagement with lifestyle modifications. A history of coronary disease often represents a patient population with more advanced or recurrent cardiovascular disease, a greater symptom burden, or a more conservative exercise progression. Consequently, these groups may benefit from intensified secondary prevention, targeted smoking cessation support, and closer monitoring of their exercise response.

Higher mean RPE was concurrently associated with suboptimal relative workload progression. Because mean RPE and workload progression were assessed over the same rehabilitation period, this association should not be interpreted as predictive or causal. Rather, higher perceived exertion may identify patients experiencing greater difficulty with workload advancement. Although direct evidence linking higher RPE to suboptimal session-level workload progression is limited, prior studies suggest that perceived exertion may not reliably reflect objective training intensity, and may be affected by factors other than physical effort [27,28,29]. Accordingly, RPE should serve not only as a guide for exercise prescription but also as a clinical marker for patients requiring closer evaluation. Persistently high RPE, particularly when evident during modest workloads, may indicate underlying issues such as deconditioning, lower cardiac output, anemia, pulmonary or musculoskeletal limitations, medication effects, chronotropic incompetence, pain or anxiety.

Lower attendance was concurrently associated with both definitions of suboptimal progression. However, given the retrospective design, the direction of this association cannot be established: fewer attended sessions may limit opportunities for progressive workload advancement, whereas poorer exercise tolerance or a more complicated clinical course may also result in lower attendance. Prior studies similarly link greater CR participation with improved oxygen consumption [30] and progressive workload advancement with gains in functional capacity [21]. Because the primary progression measure compared the first four sessions with the final four attended sessions, patients completing more sessions had a longer opportunity for workload advancement. We therefore performed a sensitivity analysis using a standardized interval, comparing sessions 1–4 with sessions 13–16 in all participants. Under this standardized definition, higher BMI and mean RPE remained associated with suboptimal relative progression, while coronary artery disease remained associated with suboptimal absolute progression. In contrast, associations with female sex were attenuated and the association with recent smoking was not reproduced. For BMI and absolute progression, the effect estimate remained similar but the confidence interval included unity. These findings indicate that some associations identified in the primary models were more robust to standardization of the progression interval than others and reinforce the need for cautious interpretation of individual covariate effects.

This study has several clinical implications. The high incidence of suboptimal workload progression underscores the need to reevaluate how exercise prescriptions are advanced in routine CR practice, as reliance on RPE or baseline exercise testing alone may be insufficient to guide individualized progression. Routinely collected CR training data may help identify patients who are not progressing adequately before program completion, allowing earlier adjustment of exercise prescriptions. The absolute and relative progression thresholds provide complementary information: The 1-MET threshold is clinically intuitive and was informed by the established prognostic relevance of 1-MET changes in measured exercise capacity; however, this evidence cannot be directly extrapolated to changes in session-level training workload. The 30% threshold accounts for baseline workload and may better capture proportional improvement among patients starting at lower exercise levels. Importantly, neither threshold has been validated against functional or clinical outcomes when applied to session-level training workload; therefore, they should be interpreted as exploratory markers of workload progression rather than established definitions of CR response. If prospectively validated, session-level workload progression could potentially complement established CR process and outcome measures such as attendance, exercise-test performance, and risk-factor control. Patient characteristics associated with inadequate workload progression included female sex, higher BMI, recent smoking, history of coronary artery disease, higher RPE, and lower attendance. Recognition of these characteristics may help guide closer monitoring and individualized evaluation during CR.

Strengths of the study include the real-world multidisciplinary setting, use of routinely recorded treadmill speed and grade to quantify workload, evaluation of absolute and relative progression, and inclusion of clinical, patient-reported, and training variables.

Limitations include the retrospective, single-center design, which precludes causal inference; the small number of women; and selection of patients who attended ≥16 sessions and were able to exercise on a treadmill. The requirement for ≥16 attended sessions and ability to exercise on a treadmill may have introduced selection bias toward more adherent and functionally able patients, limiting generalizability to patients with poorer attendance or those unable to perform treadmill exercise. Workload was estimated rather than measured by cardiopulmonary gas exchange, and other aerobic modalities and resistance training were not captured. Additional training characteristics such as duration and frequency were not fully evaluated [31]. Finally, the progression thresholds are exploratory and have not been standardized or validated for session-based CR workload progression; therefore, they should not be interpreted as established indicators of CR response. In addition, threshold-based classification dichotomizes a continuous change measure and may therefore result in loss of information. Prospective multicenter studies should validate these thresholds against directly measured changes in cardiorespiratory fitness, functional outcomes, and clinical outcomes.

## 5. Conclusions

In conclusion, supervised CR was associated with significant overall improvements in treadmill exercise workload, while more than half of patients demonstrated suboptimal progression according to both absolute and relative prespecified exploratory thresholds. In the primary multivariable analyses, female sex, higher BMI, recent smoking, and history of coronary artery disease were baseline characteristics associated with suboptimal workload progression, whereas lower attendance and higher perceived exertion were concurrent training-related factors. These associations were not materially altered after additional adjustment for initial treadmill workload. However, when workload progression was evaluated over a standardized 16-session interval, higher BMI and mean RPE remained associated with suboptimal relative progression and coronary artery disease remained associated with suboptimal absolute progression, whereas some other associations were attenuated. Collectively, these findings support the potential utility of routinely collected session-level exercise workload data for monitoring workload progression during CR, while emphasizing that the observed associations are exploratory and should not be interpreted as validated predictive markers.

## Figures and Tables

**Figure 1:**
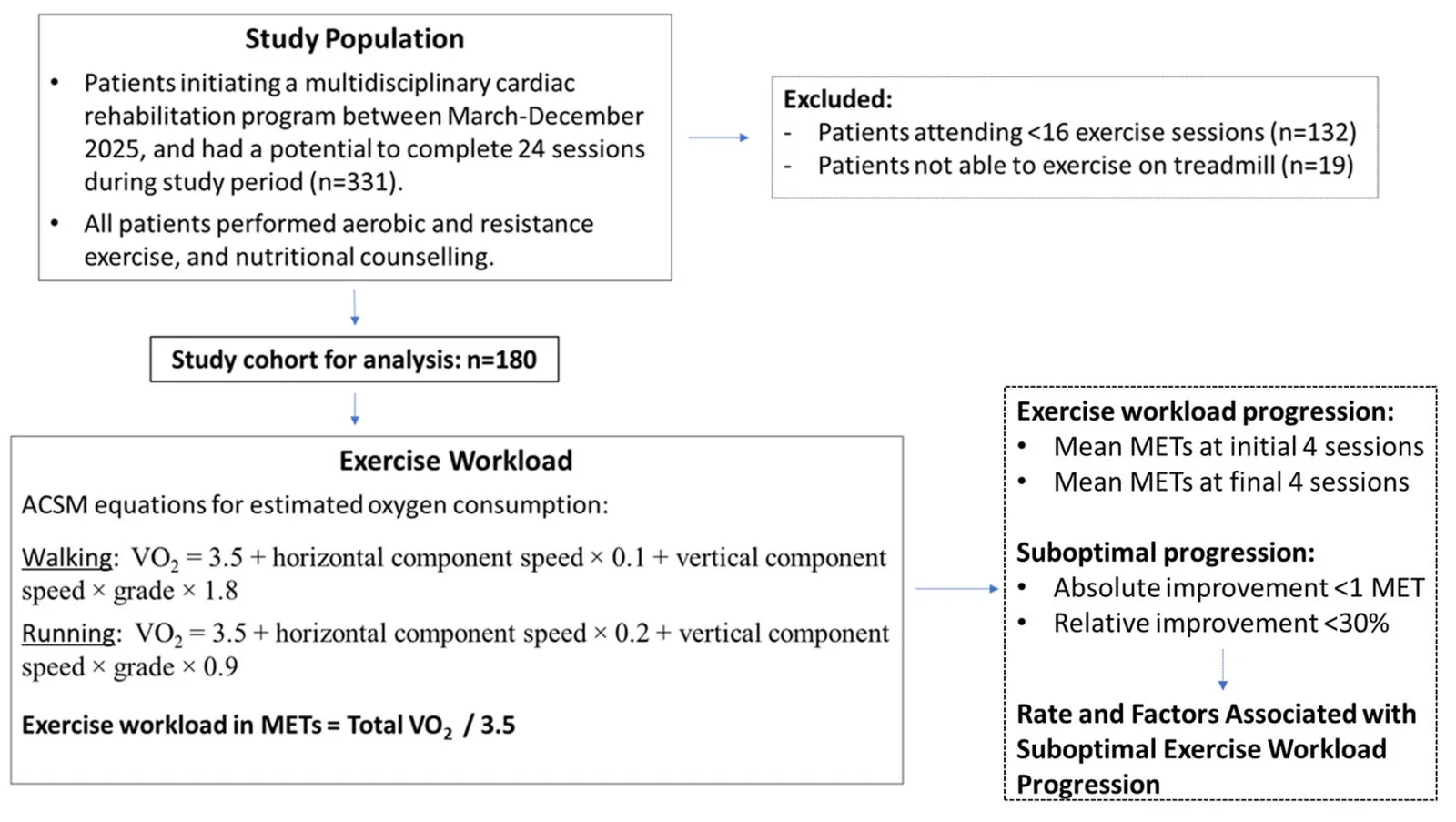
Study workflow and definition of suboptimal workload progression. Patients were eligible for 24 cardiac rehabilitation sessions; those attending fewer than 16 or unable to use a treadmill were excluded. Treadmill speed and grade were converted to estimated oxygen consumption and METs using ACSM equations. Suboptimal progression was less than a 1-MET absolute or 30% relative increase between the first and last four sessions. ACSM, American College of Sports Medicine; VO_2_, oxygen consumption; MET, metabolic equivalents.

**Figure 2:**
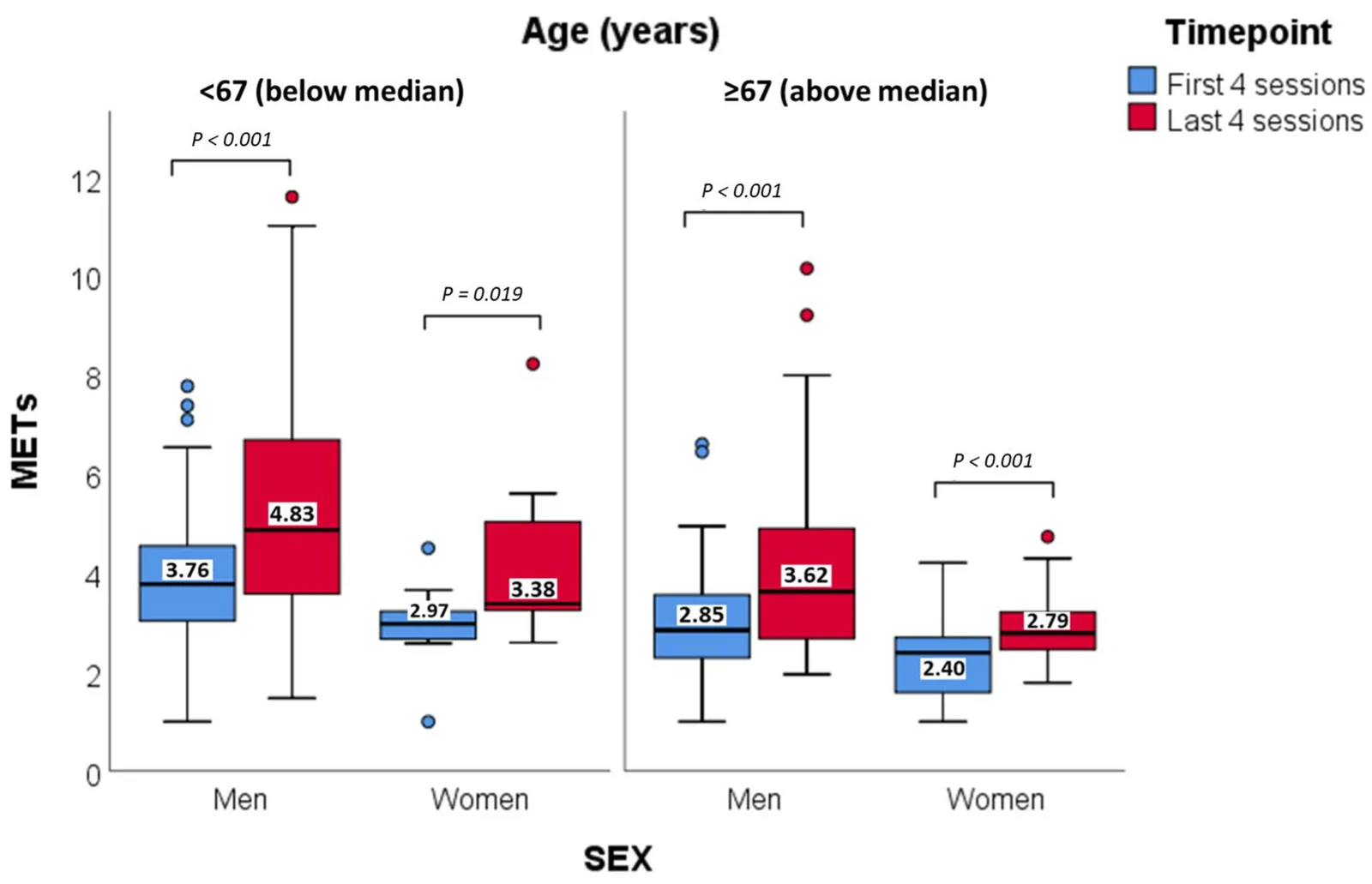
Exercise workload changes from the first to last four rehabilitation sessions by sex and age. Boxplots show significant exercise workload increases in men and women below and above the median age of 67 years, with higher median METs in men. Boxes indicate the interquartile range (IQR); lines, labeled medians; whiskers, non-outlier values within 1.5 × IQR; and dots, outliers. *P* values are paired Wilcoxon signed-rank tests comparing the first and last four sessions.

**Table 1:** Patient characteristics, according to thresholds of change in exercise workload during cardiac rehabilitation.

Variable	All Patients (n = 180)	Absolute Improvement < 1 MET (n = 104; 57.8%)	Absolute Improvement ≥ 1 MET (n = 76; 42.2%)	*p* Value	Relative Improvement < 30% (n = 97; 53.9%)	Relative Improvement ≥ 30% (n = 83; 46.1%)	*p* Value
Age, yr	67 (57–73)	67.5 (59–73)	62 (54.5–72)	0.072	67 (58–73)	66 (55–73)	0.416
Female sex	34 (18.9%)	26 (25%)	8 (10.5%)	0.014	24 (24.7%)	10 (12%)	0.030
Recent smoking	35 (19.4%)	23 (22.1%)	12 (15.8%)	0.290	24 (24.7%)	11 (13.3%)	0.052
Diabetes mellitus	66 (36.7%)	38 (36.5%)	28 (36.8%)	0.967	35 (36.1%)	31 (37.3%)	0.860
Hypertension	113 (62.8%)	66 (63.5%)	47 (61.8%)	0.824	59 (60.8%)	54 (65.1%)	0.558
Body mass index (kg/m^2^)	27.6 ± 4.7	28.2 ± 5.1	26.7 ± 3.8	0.031	28.5 ± 5.2	26.6 ± 3.8	0.007
Obesity	47 (26.1%)	32 (30.8%)	15 (19.7%)	0.096	31 (32%)	16 (19.3%)	0.053
Chronic kidney disease	37 (20.6%)	27 (26%)	10 (13.2%)	0.036	21 (21.6%)	16 (19.3%)	0.695
Hemoglobin < 12 g/dL	43 (23.9%)	26 (25%)	17 (22.4%)	0.683	24 (24.7%)	19 (22.9%)	0.772
Use of beta-blockers	157 (87.2%)	92 (88.5%)	65 (85.5%)	0.560	85 (87.6%)	72 (86.7%)	0.860
History of coronary artery disease	45 (25%)	34 (32.7%)	11 (14.5%)	0.005	29 (29.9%)	16 (19.3%)	0.101
Peripheral vascular disease	13 (7.2%)	10 (9.6%)	3 (3.9%)	0.147	9 (9.3%)	4 (4.8%)	0.249
LVEF ≤ 40%	45 (25%)	29 (27.9%)	16 (21.1%)	0.296	25 (25.8%)	20 (24.1%)	0.796
Heart failure	32 (17.8%)	24 (23.1%)	8 (10.5%)	0.030	21 (21.6%)	11 (13.3%)	0.142
Cardiac Surgery (recent)	67 (37.2%)	34 (32.7%)	33 (43.4%)	0.141	30 (30.9%)	37 (44.6%)	0.059
Myocardial infarction (recent)	69 (38.3%)	41 (39.4%)	28 (36.8%)	0.725	39 (40.2%)	30 (36.1%)	0.576
EQ-VAS Scale < 50	46 (25.6%)	26 (25%)	20 (26.3%)	0.842	25 (25.8%)	21 (25.3%)	0.942
GAD-7 ≥ 10	46 (25.6%)	28 (26.9%)	18 (23.7%)	0.623	26 (26.8%)	20 (24.1%)	0.678
PHQ-9 ≥ 10	54 (30%)	36 (34.6%)	18 (23.7%)	0.114	33 (34%)	21 (25.3%)	0.203
Low handgrip strength women < 16 kg; men < 27 kg	59 (32.8%)	36 (34.6%)	23 (30.3%)	0.539	32 (33%)	27 (32.5%)	0.948
Peak METs (admission exercise test)	5.9 (3.3–8.0)	5.6 (3.3–7.1)	6.4 (3.3–9.4)	0.114	5.6 (3.5–7.3)	6.1 (3.1–8.9)	0.660
Number of attended sessions (range 16–24)	24 (22–24)	24 (21–24)	24 (23–24)	0.110	24 (21–24)	24 (23–24)	0.321
Average RPE (Borg scale)	12.4 ± 1.3	12.6 ± 1.3	12.1 ± 1.2	0.031	12.5 ± 1.3	12.2 ± 1.2	0.061

Data are presented as number (percentage), mean and standard deviation or median (interquartile range), according to distribution. Thresholds represent absolute change of 1 MET or relative change of 30% between mean METs during the initial and final four CR sessions. EQ-VAS, EuroQol-visual analogue scale (self-perceived health); GAD-7, generalized anxiety disorder-7 questionnaire; LVEF, left ventricular ejection fraction; METs, metabolic equivalents; PHQ-9, patient Health Questionnaire-9; RPE, rating of perceived exertion.

**Table 2:** Exercise workload progression during cardiac rehabilitation and rates of suboptimal progression, according to admission peak METs achieved at exercise testing.

Admission Peak METs (Exercise Testing)	Initial Four Sessions METs	Final Four Sessions METs	Improvement <1 MET	Relative Improvement <30%
<5 METs (n = 69; 38.3%)	2.29 (1.71–2.79)	2.93 (2.49–3.55)	43 (62.3%)	38 (55.1%)
5–8 METs (n = 67; 37.2%)	3.23 (2.87–3.70)	4.24 (3.38–5.03)	42 (62.7%)	39 (58.2%)
>8 METs (n = 44; 24.4%)	4.49 (4.06–5.72)	6.57 (5.32–8.11)	19 (43.2%)	20 (45.5%)
Overall (n = 180)	3.07 (2.40–4.01)	3.90 (2.93–5.56)	104 (57.8%)	97 (53.9%)

Values are presented as median (interquartile range) or number (percentage). METs, metabolic equivalents. *p* < 0.001 for all comparisons between the initial and final four sessions.

**Table 3:** Univariate odds ratios for suboptimal workload progression during cardiac rehabilitation.

Variable	OR (95% CI) for Improvement < 1 MET	*p* Value	OR (95% CI) for Relative Improvement < 30%	*p* Value
Age, yr	1.027 (0.998–1.057)	0.068	1.01 (0.98–1.04)	0.456
Female Sex	2.83 (1.20–6.67)	0.017	2.40 (1.07–5.37)	0.033
Recent smoking	1.51 (0.70–3.27)	0.291	2.15 (0.98–4.72)	0.056
Diabetes mellitus	0.99 (0.53–1.82)	0.967	0.95 (0.52–1.74)	0.860
Hypertension	1.07 (0.58–1.98)	0.824	0.83 (0.45–1.53)	0.558
Body mass index (kg/m^2^)	1.08 (1.01–1.15)	0.034	1.10 (1.02–1.17)	0.008
Obesity	1.81 (0.90–3.65)	0.098	1.97 (0.98–3.93)	0.056
Chronic kidney disease	2.31 (1.04–5.13)	0.039	1.16 (0.56–2.39)	0.695
Hemoglobin < 12 g/dL	1.16 (0.58–2.33)	0.683	1.11 (0.56–2.21)	0.772
Use of beta-blockers	1.30 (0.54–3.12)	0.561	1.08 (0.45–2.60)	0.860
History of coronary disease	2.87 (1.34–6.13)	0.006	1.79 (0.89–3.59)	0.103
Peripheral vascular disease	2.59 (0.69–9.75)	0.160	2.02 (0.60–6.82)	0.257
LVEF ≤ 40%	1.45 (0.72–2.92)	0.297	1.09 (0.56–2.16)	0.796
Heart failure	2.55 (1.08–6.04)	0.034	1.81 (0.82–4.02)	0.145
Cardiac Surgery (recent)	0.63 (0.34–1.17)	0.142	0.56 (0.30–1.03)	0.060
Myocardial infarction (recent)	1.12 (0.61–2.05)	0.725	1.19 (0.65–2.17)	0.577
EQ-VAS Scale <50	0.93 (0.47–1.84)	0.842	1.03 (0.52–2.01)	0.942
GAD-7 ≥ 10	1.19 (0.60–2.35)	0.623	1.15 (0.59–2.26)	0.678
PHQ-9 ≥ 10	1.71 (0.88–3.32)	0.116	1.52 (0.80–2.91)	0.204
Low handgrip strength (<16 kg women; <27 kg men)	1.22 (0.65–2.30)	0.539	1.02 (0.55–1.91)	0.950
Peak achieved METs (admission exercise test)	0.94 (0.86–1.02)	0.150	0.98 (0.90–1.07)	0.698
Number of attended sessions (range 16–24)	0.89 (0.78–1.01)	0.074	0.88 (0.77–1.002)	0.053
Average RPE (Borg scale)	1.31 (1.02–1.67)	0.033	1.26 (0.99–1.60)	0.064

CI, confidence interval; EQ-VAS, EuroQol visual analogue scale; GAD-7, Generalized Anxiety Disorder-7 questionnaire; LVEF, left ventricular ejection fraction; METs, metabolic equivalents; OR, odds ratio; PHQ-9, Patient Health Questionnaire-9; RPE, rating of perceived exertion.

**Table 4:** Final multivariable models showing factors associated with suboptimal workload progression during cardiac rehabilitation.

Variable	Adjusted OR (95% CI)	*p* Value
Model 1: <30% relative improvement		
Female sex	2.64 (1.04–6.67)	0.040
Body mass index, per 1 kg/m^2^ increase	1.11 (1.03–1.19)	0.009
Recent smoking	2.55 (1.02–6.35)	0.045
Attended sessions (range, 16–24)	0.86 (0.75–0.998)	0.048
Mean RPE (Borg scale)	1.36 (1.03–1.79)	0.028
Model 2: <1 MET absolute improvement		
Female sex	3.19 (1.1–8.43)	0.019
Body mass index, per 1 kg/m^2^ increase	1.08 (1.00–1.17)	0.046
History of coronary artery disease	2.48 (1.02–6.04)	0.046
Attended sessions (range, 16–24)	0.83 (0.72–0.96)	0.011

Model 1: Omnibus *p* = 0.020; Nagelkerke R2 = 0.225; Hosmer–Lemeshow *p* = 0.166. Model 2: Omnibus *p* = 0.015; Nagelkerke R2 = 0.250; Hosmer–Lemeshow *p* = 0.430. CI, confidence interval; OR, odds ratio; RPE, rating of perceived exertion.

## Data Availability

The data are available from the corresponding author upon reasonable request.

## Supplementary Materials

The following supporting information can be downloaded at https://www.mdpi.com/article/10.3390/jcm15197713/s1. Table S1: Multivariable models of factors associated with suboptimal workload progression after additional adjustment for mean METs during the initial four session; Table S2: Multivariable models of factors associated with suboptimal workload progression using a standardized session interval (sessions 1–4 versus sessions 13–16); Supplementary Data, case descriptions illustrating exercise workload progression during cardiac rehabilitation.
