# Supplementary material for: Incidence and Factors Associated with Suboptimal Exercise Workload Progression in Cardiac Rehabilitation: A Retrospective Cohort Study
Source: J Clin Med. 2026 Oct 6;15(19):7713. doi: 10.3390/jcm15197713 (PMC13665919; doi:10.3390/jcm15197713)
Supplement: Supplementary file 1 [file jcm-15-07713-s001.zip › jcm-4572019-supplementary.pdf]

Supplementary Materials

Table S1. Multivariable models showing factors associated with suboptimal workload progression after additional adjustment for mean METs during the initial four sessions.

Variable	Adjusted OR (95% CI)	P value
**Model 1: <30% relative improvement**		
Female sex	2.82 (1.10-7.23)	0.031
Body mass index, per 1 kg/m2 increase	1.11 (1.02-1.19)	0.010
Recent smoking	2.60 (1.04-6.52)	0.041
Attended sessions (range, 16-24)	0.86 (0.74-0.99)	0.046
Mean RPE (Borg scale)	1.37 (1.04-1.81)	0.025
**Model 2: <1 MET absolute improvement**		
Female sex	3.47 (1.31-9.22)	0.012
Body mass index, per 1 kg/m2 increase	1.09 (1.01-1.17)	0.037
History of coronary artery disease	2.47 (1.01-6.06)	0.048
Attended sessions (range, 16-24)	0.85 (0.73-0.99)	0.037

CI, confidence interval; OR, odds ratio; RPE, rating of perceived exertion.

Table S2: Multivariable models of factors associated with suboptimal workload progression using a standardized session interval (sessions 1–4 versus sessions 13–16).

Variable	Adjusted OR (95% CI)	P value
Model 1: <30% relative improvement		
Female sex	1.88 (0.78-4.53)	0.163
Body mass index, per 1 kg/m^2 increase	1.09 (1.01-1.17)	0.025
Recent smoking	1.21 (0.53-2.75)	0.656
Mean RPE (Borg scale)	1.36 (1.05-1.76)	0.020
Model 2: <1 MET absolute improvement		
Female sex	2.27 (0.90-5.75)	0.084
Body mass index, per 1 kg/m^2 increase	1.08 (0.99-1.17)	0.071
History of coronary artery disease	3.90 (1.51-10.05)	0.005

CI, confidence interval; OR, odds ratio; RPE, rating of perceived exertion.

Supplementary Data: Case Descriptions Illustrating Exercise Workload Progression during Cardiac Rehabilitation

Suboptimal workload progression was defined as an absolute increase of <1 MET and/or a relative increase of <30% between the mean workload of the first four and last four rehabilitation sessions.

Case 1. Suboptimal absolute and relative workload progression

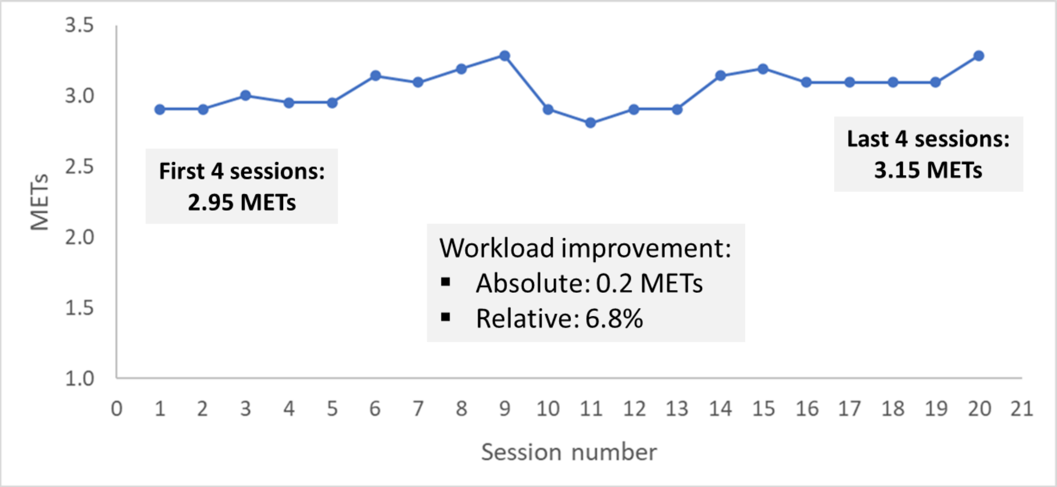

A 67-year-old man was admitted to cardiac rehabilitation one month after transcatheter aortic valve replacement (TAVR). The patient's cardiovascular risk factors included type 2 diabetes, hypertension, and chronic kidney disease. He had coronary artery disease with previous coronary artery bypass graft (CABG) surgery as well as heart failure with reduced ejection fraction. His left ventricular ejection fraction (LVEF) was 30%. He also suffered from peripheral artery disease. During the admission symptom-limited treadmill exercise test, he achieved a peak of 5.2 METs.

Case 2. Suboptimal absolute and relative workload progression

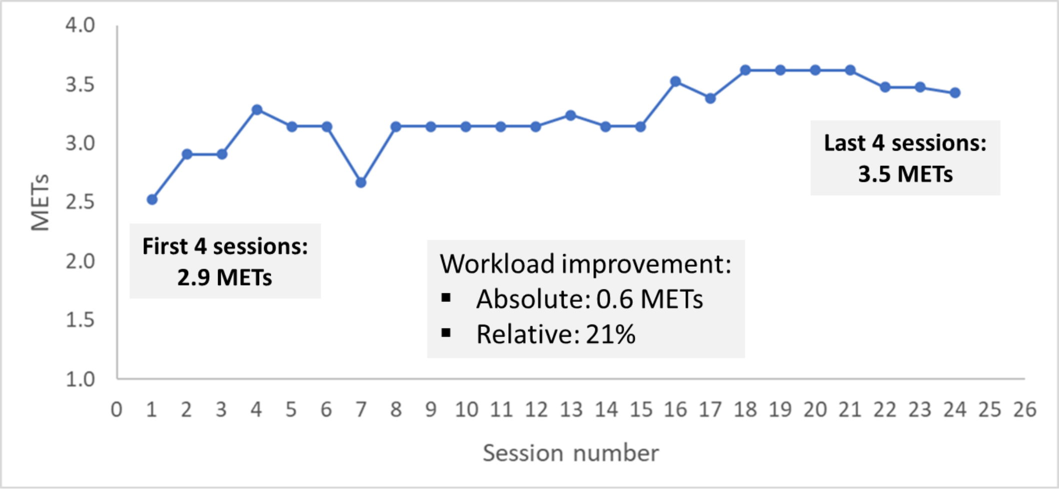

A 75-year-old woman with a history of smoking, hypertension, hyperlipidemia, obesity and obstructive sleep apnea was referred to cardiac rehabilitation one month after coronary revascularization. She had initially presented with chest pain and evidence of myocardial ischemia on a perfusion scan. Coronary angiography revealed multivessel coronary artery disease, and percutaneous coronary intervention (PCI) was performed with drug-eluting stent implantation in the left anterior descending and right coronary arteries; the circumflex artery was chronically occluded. Echocardiography showed preserved LVEF with a segmental wall motion abnormality of the inferior wall. At

admission to cardiac rehabilitation, symptom-limited treadmill exercise testing was terminated at an early stage because of dyspnea and lower-limb pain.

Case 3. Suboptimal absolute but adequate relative workload progression

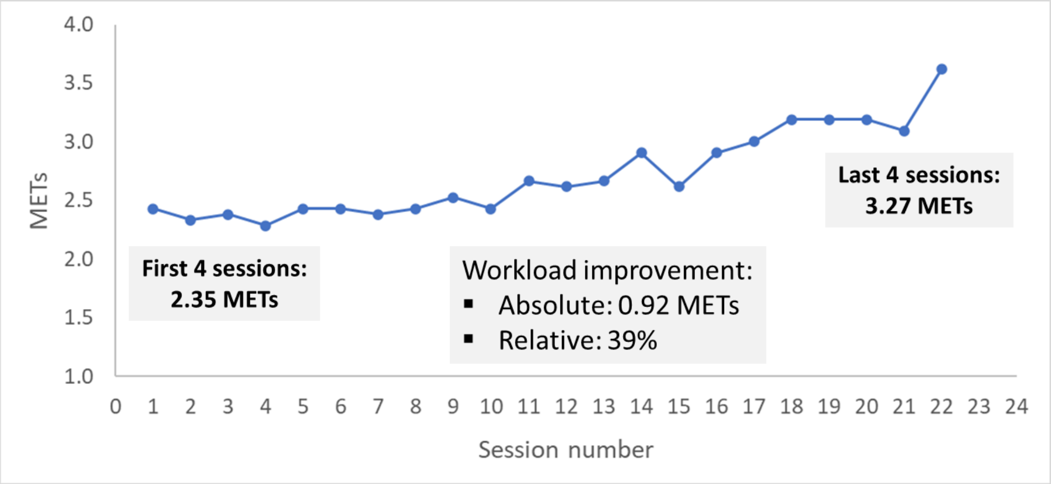

A 60-year-old diabetic man was admitted to the cardiac intensive care unit after out-of-hospital ventricular fibrillation cardiac arrest secondary to an acute anterolateral myocardial infarction. Echocardiography on admission revealed severe aortic valve stenosis with mildly reduced left ventricular function (LVEF 45%). The patient underwent cardiac surgery consisting of CABG and aortic valve replacement. Two months after hospital discharge, he initiated cardiac rehabilitation. He achieved a peak of 5.6 METs during the admission symptom-limited treadmill exercise test.

Case 4. Adequate absolute and relative workload progression

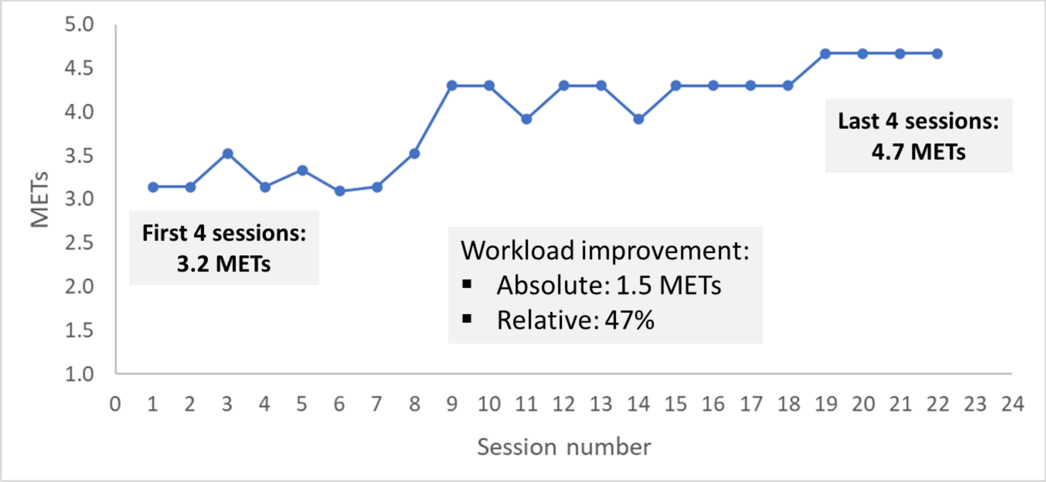

A 77-year-old man with cardiovascular risk factors including type 2 diabetes, hypertension, prior smoking and chronic kidney disease was admitted to cardiac rehabilitation. He had known coronary artery disease with prior myocardial infarction, and a preserved LVEF. In addition, he suffered from intermittent claudication secondary to peripheral artery disease. He achieved a peak of 5.1 METs during admission treadmill exercise test, which was limited by leg pain when walking.

Case 5. Adequate absolute and relative workload progression

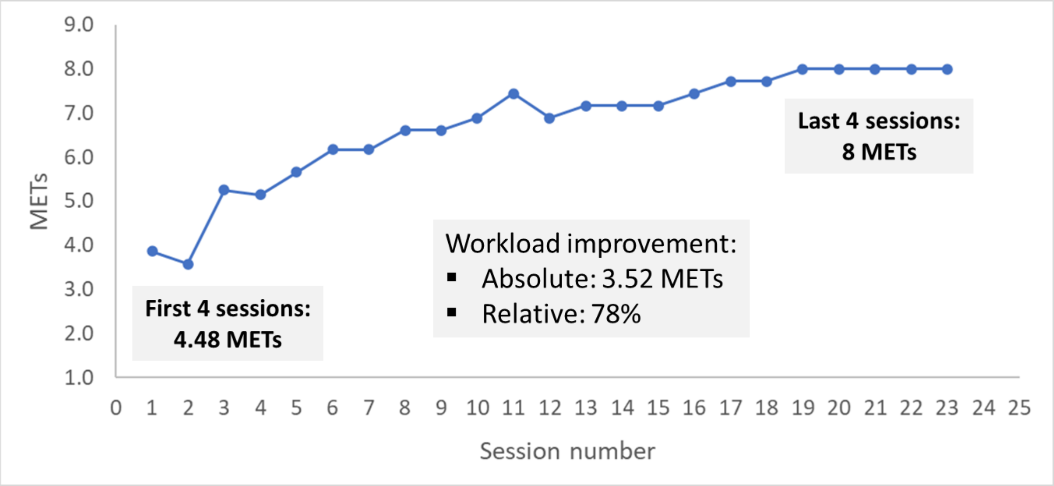

A 77-year-old physically active man with type 2 diabetes and chronic kidney disease was hospitalized due to acute non-ST-segment elevation myocardial infarction. Coronary angiography revealed multivessel coronary artery disease. An echocardiogram showed moderately reduced left ventricular function (LVEF 40%). The patient was referred for a CABG, which was performed without postoperative complications. Six weeks after discharge, the patient initiated cardiac rehabilitation. He achieved a peak of 9.2 METs during the admission treadmill exercise test.
